# Comparative analysis of patients with upper urinary tract urothelial carcinoma in black-foot disease endemic and non-endemic area

**DOI:** 10.1186/s12885-021-07799-4

**Published:** 2021-01-19

**Authors:** Che-Wei Chang, Chien-Hui Ou, Chih-Chin Yu, Chi-Wen Lo, Chung-You Tsai, Pai-Yu Cheng, Yung-Tai Chen, Hsu-Che Huang, Chia-Chang Wu, Ching-Chia Li, Hsiang-Ying Lee

**Affiliations:** 1 Department of Urology, Kaohsiung Medical University Chung Ho Memorial Hospital, Kaohsiung, Taiwan; 2 Department of Urology, National Cheng-Kung University Hospital, Tainan, Taiwan; 3 Department of Urology, Taipei Tzu Chi Hospital, Buddhist Tzu Chi Medical Foundation, New Taipei City, Taiwan; 4 Department of Urology, Far-Eastern Memorial Hospital, New Taipei City, Taiwan; 5 Department of Urology, Taiwan Adventist Hospital, Taipei, Taiwan; 6 Department of Urology, Cardinal Tien Hospital, New Taipei City, Taiwan; 7 Department of Urology, Shuang Ho Hospital, Taipei Medical University, New Taipei City, Taiwan; 8 Department of Urology, Kaohsiung Municipal Ta-Tung Hospital, Kaohsiung, Taiwan

**Keywords:** Arsenic, Upper urinary tract urothelial carcinoma, Black foot disease, Clinicopathological features, Prognosis

## Abstract

**Background:**

A high incidence of upper urinary tract urothelial carcinoma has been reported in the southwestern area of Taiwan, where arsenic water contamination was considered the main cause. However, there is no definite proof to show a correlation between arsenic water contamination and upper urinary tract urothelial carcinoma. To investigate the clinical and epidemiological features of patients with upper urinary tract urothelial carcinoma between arsenic water endemic and non-endemic areas, we analyzed patients in terms of characteristics, stratified overall survival, disease-free survival, and cancer-specific survival.

**Methods:**

The records of a total of 1194 patients diagnosed with upper urinary tract urothelial carcinoma were retrospectively reviewed. Clinical data and current medical status were collected from the medical records. Statistical analyses were performed to determine the clinical variables and stratified survival curves between endemic and non-endemic groups.

**Results:**

Female predominance was revealed in both endemic and non-endemic groups (male:female ratio = 1:1.2–1.4). No statistical differences were found in histological types, staging, and tumor size between the two groups. Nonetheless, patients with characteristics of aging and having end-stage renal disease were outnumbered in the non-endemic group, while a higher prevalence of previous bladder tumors and more ureteral tumors were found in the endemic group. Adjusted stratified cumulative survival curves suggested a poorer prognosis in endemic patients, especially in disease-free survival of early stage disease.

**Conclusions:**

A higher mortality rate with more previous bladder cancer history and ureteral tumors was seen in patients with upper urinary tract urothelial carcinoma residing in the arsenic water contamination area. This may be attributed to the long-term carcinogenic effect of arsenic underground water.

## Background

Among urothelial tumors, upper urinary tract urothelial carcinoma (UTUC) in the renal pelvis only accounts for 5% of all [1]. The incidence of UTUC located at the ureter is less common than that of renal pelvis tumors, with a ratio of 1:3 to 1:4 [2]. In previous studies, an unusually high incidence of urothelial carcinoma, including bladder, ureter, and renal pelvis tumors, was identified in Taiwan, but the risk factors remain controversial [3].

Long-term exposure to arsenic underground water has been an issue in many countries, including Finland, Argentina, Chile, and Taiwan [4–7]. It could lead to black-foot disease (BFD), a peripheral vascular disease that results in dry gangrene of the affected extremities [8]. Higher adjusted odds ratios of developing bladder, lung, and liver cancers were also observed in arsenic-exposed residents in southwestern Taiwan [4]. Similar cases have been reported in lower urinary tract cancers and kidney cancers. Cumulative mortality rates were also higher in the BFD-endemic area than in the general population [9]. Additionally, smokers with high arsenic exposure had increased risks of 5.7 and 6.4 for bladder cancer and UTUC, respectively [10].

One recent study investigated genitourinary (GU) tract cancer patients exposed to an arsenic environment. The total number of patients was 474, including 328 patients with bladder cancer and 146 patients with UTUC. A significantly higher mortality rate for urinary bladder cancers and an unusually high incidence of UTUC in the BFD endemic area were concluded [11]. Nevertheless, the limited number of UTUC patients exposed to arsenic may confer bias. Here, we retrospectively reviewed 1194 patients with pathologically confirmed UTUC to clarify the epidemiological and prognostic differences in endemic and non-endemic BFD areas.

## Methods

### Patients

From July 1988 to March 2019, the medical records of 1194 patients with pathologically proven UTUC at eight hospital centers (National Cheng Kung University Hospital, Kaohsiung Medical University Hospital, Taipei Tzu Chi Hospital, Far Eastern Memorial Hospital, Taiwan Adventist Hospital, Yonghe Cardinal Tien Hospital, Taipei Medical University-Shuang Ho Hospital, and Taiwan University Hospital) were reviewed. No experiments on humans were conducted, and thus informed consent and guidelines were not required. This study was approved by all eight institutional review boards (National Cheng Kung University Hospital, Kaohsiung Medical University Hospital, Taipei Tzu Chi Hospital, Far Eastern Memorial Hospital, Taiwan Adventist Hospital, Yonghe Cardinal Tien Hospital, Taipei Medical University-Shuang Ho Hospital, and Taiwan University Hospital [KMUHIRB-E (I)-20,180,214]). The requirement for consent and guidelines was waived by institutional review boards. Clinical variables include age, sex, residency in black-foot-disease (BFD) endemic or BFD non-endemic area, tumor size (pathological), tumor location, and tumor grade according to the standard WHO grading system. Tumor grade 1 in the 1973 WHO grading system [12] refers to low-grade tumors in the 2004 WHO grading system [13] while grade 2 and grade 3 tumors refer to high-grade tumors. Pathological tumor stage is based on specimens obtained from nephroureterectomy with or without lymph node dissection according to the TNM (tumor, nodes and metastases) system [14]. Other patient characteristics such as smoking, end-stage renal disease, and history of urothelial carcinoma in the bladder were also included. BFD endemic area was defined as southwestern Taiwan, including four towns in the core zone (Budai, Yijhu, Beimen, and Syuejia) and the peripheral area surrounding the core zone (Yun-Lin, Chia-Yi, Tainan, Kaohsiung, and Pingtung) [15]. BFD non-endemic area was defined as northern Taiwan. Residency is based on the personal information provided by the patients during hospitalization. We also prospectively examined tumor recurrence, tumor progression, and prognosis until May 2019.

### Statistical analyses

Pearson’s chi-square test was used for univariate analysis for all clinical variables. For independent clinical factors associated with endemic areas, multivariate logistic regression analysis was performed based on significant variables from univariate analysis. Survival estimates were obtained using Kaplan-Meier survival curves with log-rank test stratified by pathological tumor stages. All statistical tests with *p*-values lower than 0.05 were considered significant.

## Results

In total, 527 patients from non-endemic areas and 667 patients from endemic areas were retrospectively analyzed. Overall, female predilection of 1:1.29 was observed but there was no significant difference between non-endemic and endemic groups. A higher proportion (51.7%) of the aged group was observed in the non-endemic group than in the endemic group (39.2%). More end-stage renal disease patients were found in the non-endemic group, while more patients with bladder cancer history were noted in the endemic group. There were no significant differences between the two groups in smoking, non-urothelial carcinoma cancer, ureteral tumor, tumor size, or tumor grade. However, the endemic group had a significantly lower proportion of renal pelvis tumors and higher proportion of ureteral tumors (Table 1). As for treatment options, most of the patients received nephroureterectomy with bladder cuff excision in both groups (79.6% in the non-endemic group and 90.4% in the endemic group). Others received segmental resection and endoscopic ablation. There was a low proportion of patients who did not undergo any operation. No significant differences were noted in preoperative renal function between the groups. Adjuvant chemotherapy was observed in 10% of the non-endemic group and 9.1% in the endemic group.

**Table 1:** Demographic characteristics of UTUC patients in black-foot-disease (BFD) non-endemic and endemic area

Characteristics	Non-endemic *N* = 527	Endemic *N* = 667	*P*-value
**Age**			
< 50	29	47	< 0.001
50 ~ 59	74	111	
60 ~ 69	139	210	
> 70	259	237	
**Sex**			
Male	222	298	0.377
Female	305	369	
**Smoking**			
Yes	192	251	0.865
No	69	41	
**ESRD**			
Yes	105	63	< 0.001
No	156	223	
**Non UC tumor**			
Yes	54	46	0.063
No	469	602	
**Previous bladder tumor**			
Yes	30	54	< 0.017
No	231	232	
**Tumor location**			
Renal pelvis	369	367	< 0.001
Upper ureter	111	117	0.241
Middle ureter	60	98	0.049
Distal ureter	103	140	0.317
Bladder cuff	7	13	0.354
**Tumor size**			
non-visible	7	3	0.138
< 1 cm	87	22	
≥ 1 & < 2 cm	95	43	
≥ 2 & < 3 cm	98	45	
≥ 3 cm	214	108	
**Tumor grade**			
low grade	63	44	0.157
high grade	340	320	
**Pathologic staging**			
stage 0a/0is	43	113	< 0.001
stage I	106	150	
stage II	112	119	
stage III	125	137	
stage IV	30	98	
**Treatment options**			
NxUx	420	603	0.001
Segmental resection	1	14	
Endoscopic	86	9	
Adjuvant chemotherapy	53	61	0.153
No operation	22	41	
Pre-op mean renal function (eGFR)	37.185	39.216	0.87

Multivariate analyses of clinical characteristics between the groups revealed significant differences in age, end-stage renal disease, previous bladder cancer, pathological stage, tumor in renal pelvis or mid-ureter, and lymphovascular invasion (Table 2). Younger age (OR 0.974), lower proportion of end-stage renal disease (OR 0.42), higher proportion of previous bladder cancer (OR 1.792), less early-stage disease I-III (OR 0.538, 0.404, and 0.417, respectively), fewer renal pelvis tumors (OR 0.565) with more mid-ureter tumors (OR 1.416), and less lymphovascular invasion (OR 0.231) were independently observed in the BFD endemic group.

**Table 2:** Multiple variate analyses of UTUC patients’ characteristics in black-foot-disease (BFD) non-endemic and endemic area

Characteristics	Non-endemic	Endemic	OR	95CI		*P*-value
	*N* = 527	*N* = 667				
**Sex**						
male	222	298	1	–	–	
female	305	369	0.899	0.714	1.132	0.365
**Age**			0.974	0.963	0.984	< 0.001
< 50	29	47				
50 ~ 59	74	111				
60 ~ 69	139	210				
> 70	259	237				
**ESRD**						
NO	156	223	1	–	–	
YES	105	63	0.420	0.289	0.610	< 0.001
**previous bladder UC**						
NO	231	232	1	–	–	
YES	30	54	1.792	1.107	2.902	0.018
**Pathologic staging**						
stage 0a/0is	43	113	1	–	–	
stage I	106	150	0.538	0.350	0.828	0.005
stage II	112	119	0.404	0.261	0.625	< 0.001
stage III	125	137	0.417	0.272	0.639	< 0.001
stage IV	30	98	1.243	0.725	2.131	0.429
**Tumor grade**						
low grade	63	44	1	–	–	–
high grade	340	320	1.348	0.891	2.039	0.158
**Tumor location**						
Renal pelvis	369	367	0.565	0.441	0.725	< 0.001
Proximal ureter	111	117	0.842	0.630	1.126	0.246
Middle ureter	60	98	1.416	1.003	1.999	0.048
Distal ureter	103	140	1.160	0.871	1.544	0.310
Bladder cuff	7	13	1.547	0.613	3.907	0.356
**Multiple lesions**						
Not available	1	13	1	–	–	–
No	284	403	0.014	0.837		0.109
Yes	216	192	0.009	0.528		0.068
**Lymphovascular invasion**						
No	254	493	1	–	–	–
Yes	105	47	0.231	0.158	0.336	< 0.001

Follow-up disease status in both groups of UTUC patients is shown in Table 3. Disease-free rates were 38.5 and 48.27% in non-endemic and endemic groups, respectively. There was a significant difference in mean follow-up time between the non-endemic and endemic groups at 20 and 26 months, respectively. The time from operation to death was 17 months and 23.5 months for the non-endemic and endemic groups, respectively, although the difference was not statistically significant.

**Table 3:** Follow-up status of UTUC patients’ characteristics in black-foot-disease (BFD) non-endemic areas

	Non-endemic	Endemic	*P*-value
	*N* = 527	*N* = 667	
**Disease free**			
No (NxUx)	60	166	< 0.001
Yes	203	322	
No (endoscopic ablasion surgery)	47	3	
No (Segmental resection)	0	8	
Nonknown	36	32	
No	0	15	
**Mortality**			
No	181	200	< 0.001
UTUC related	39	106	
non-UTUC related	35	87	
Nonknown (Lost Follow up)	86	148	
Surgery related	9	0	
**Lost follow-up**			
No	254	414	< 0.001
Yes	259	189	
**Follow-up/month**			
Median (month)	20 (7–45)	26 (9.3–52)	0.001
**Time from operation to death**			
Median (month)	17 (5.2–37.7)	23.5 (8–43.5)	0.147

**Fig. 1:**
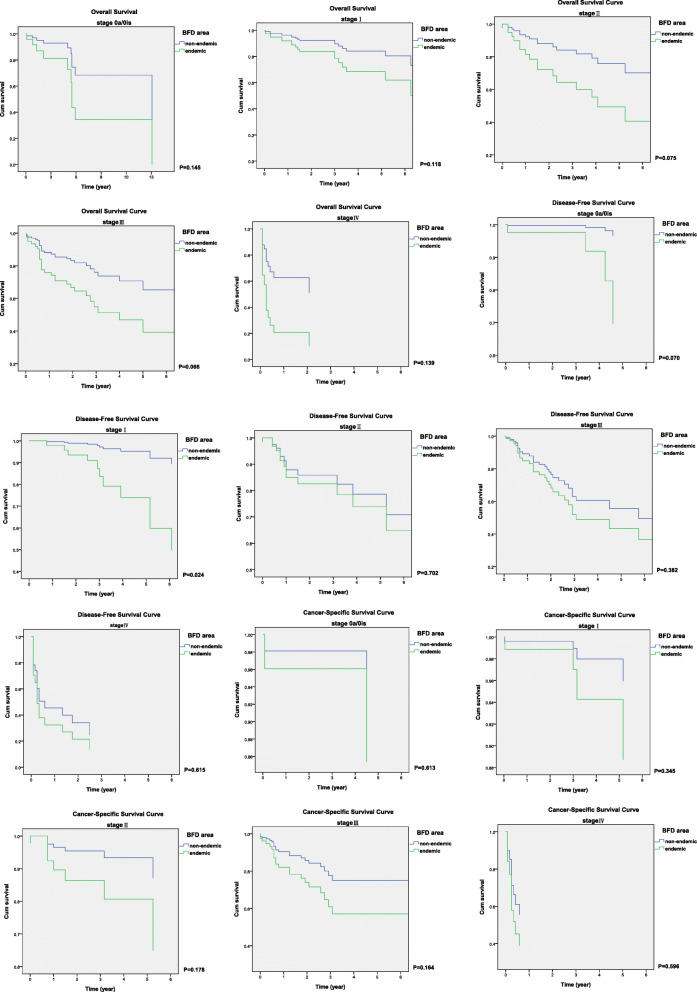
Adjusted Kaplan-Meier estimated overall survival, disease free survival and cancer specific survival curves of upper urinary tract urothelial carcinoma in stratified pathological tumor stages, non-endemic group (blue) and endemic group (green)

**Fig. 2:**
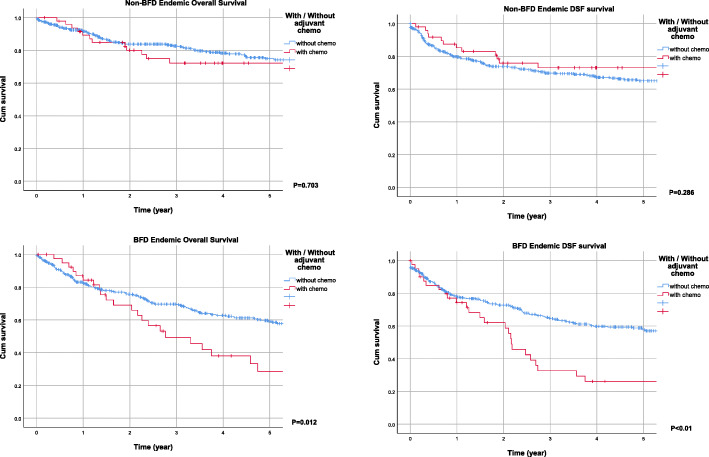
Adjusted Kaplan-Meier estimated overall survival and disease-free survival curves of upper urinary tract urothelial carcinoma with (red) or without (blue) adjuvant chemotherapy in the non-endemic area and endemic areas

In stratified pathological stages from Ta to T4 obtained from nephroureterectomy, the Kaplan-Meier estimated overall survival (OS), disease-free survival (DFS), and cancer-specific survival (CSS) curves of UTUC patients in non-endemic and endemic groups are depicted in Fig. 1. Cumulative survival rates suggested poorer prognosis in all stages of UTUC in BFD endemic areas with adjusted age, sex, tumor location, end-stage renal disease and even in patients received adjuvant chemotherapy Fig. 2. There is a significant difference in the early stages of cancer-specific survival curves.

## Discussion

UTUC is a relatively rare tumor, but the incidence and mortality rates are gradually increasing [16]. Raman JD found that a slowly increasing incidence of UTUC with increasing ureteral disease and decreasing renal pelvis or local diseases has been recorded over the past 30 years in the US. In addition, the incidence rate of UTUC involving the renal pelvis and ureter remained low in Western countries, with approximately 2.06 cases per 100,000 person-years, which accounts for 5% of all urothelial carcinomas [17, 18]. However, the percentage of renal pelvis and ureter urothelial carcinoma accounts for 20% of all urothelial carcinomas in southern Taiwan [19], suggesting a high prevalence of UTUC in Taiwan.

At diagnosis, tumors in the renal pelvis are two to three times more common than in the ureter [17, 20]. Nevertheless, the incidence rate of ureteral tumors was greater in Taiwan. In our study, renal pelvis tumors and ureteral tumors accounted for 70 and 30% of BFD non-endemic areas, respectively. In BFD endemic areas, 55% of UTUCs were located in the renal pelvis and 45% were located in the ureter. This is consistent with previous studies [11, 19], where the ratio of renal pelvic and ureteric tumors is around 1:1.

It has been reported that a male predilection of UTUC could be seen in other countries with a male to female ratio of approximately 2:1 and a median age of 66 to 70 years [21, 22]. However, in Taiwan, female predominance, with a male-to-female ratio of 1:2, has been reported in previous studies [11, 19].

In this study, female predominance could also be seen in both BFD non-endemic and endemic areas. The male-to-female ratio was 1:1.2–1.4, and the median age was 66 to 69. Tan [11] proposed that women in BFD-endemic areas tended to be exposed to arsenic well water due to farming, fishery, salt production, and daily laundry. However, compared to past figures, the number of female patients with UTUC decreased. Public awareness of arsenic intoxication has been aroused, which might lead to a reduction in the usage of arsenic well water. This could possibly explain the decline in female predilection in the present study.

In addition, there were no statistical differences in the proportion of smokers between the two groups. Although only half of the medical records included these data, we assumed that the variation is negligible among non-endemic and endemic groups. In addition, the percentage of female smoker in Taiwan is relatively low compared to other countries. Thus, the influence of gender difference is relatively low (male-to-female ratio is 10.9 to 1) [23]. As for incidence of non-urothelial tumors, no obvious differences were observed among two groups. The clinicopathological differences between BFD non-endemic and endemic groups in UTUC revealed higher grade and higher pathologic stages in the BFD-endemic group. This contradicts the results of previous studies where no remarkable differences in UTUC were associated with arsenic exposure. Nevertheless, a higher histologic grade was observed in urinary bladder urothelial carcinomas [11]. The higher prevalence of previous bladder tumor and ureteral tumor in the endemic BFD group suggests that urinary bladder and ureter are more sensitive to inorganic arsenic than the renal pelvis. A greater proportion of end-stage renal disease in the BFD non-endemic group could be another risk factor for developing UTUC, and a high incidence of UTUC was found in the end-stage renal disease population in Taiwan [24].

Variables of age, end-stage renal disease, and tumor location were used in adjustment of all Kaplan-Meier curves. Compared to non-endemic patients, poor prognosis of UTUC patients in BFD endemic areas was observed in terms of overall survival, disease-free survival, and cancer-specific survival curves. Poorer survival curves were found at higher pathological stages. This is compatible with a previous study showing that pathological stage is a predictive factor of prognosis in UTUC patients [25]. We also analyzed the DFS and OS between the two groups with or without adjuvant chemotherapy. It is interesting that no statistical differences of DFS and OS in the BFD non-endemic areas. However, statistical differences of DFS and OS were found in the BFD endemic areas. More poor survival rate can be seen in patients receiving adjuvant chemotherapy in the BFD endemic areas. It is mostly because patients receiving adjuvant chemotherapy are more likely to have advanced diseases. Although the prognosis of BFD endemic UTUC patients is poor, the average follow-up time was 26 months, and the average time from operation to death was 23.5 months, compared to the non-endemic group, at 20 and 17 months respectively, longer than that in the endemic group. Such differences could be attributed to arsenic intoxication; however, other confounding factors such as rural-urban disparity and latency of initial diagnosis should not be neglected. Further investigation excluding confounding factors is required to explore the prognostic indicators in UTUC patients.

Arsenic intoxication has been identified as a risk factor for lung cancer and bladder cancer in a dose-response relationship [26]. Several types of arsenic carcinogenesis have been proposed, including generation of oxidative stress, perturbation of DNA methylation patterns, inhibition of DNA repair, and modulation of signal transduction pathways [27]. Inorganic arsenic is detoxicated via a methylation process and transformed into monomethylarsonic acid (MMAV), monomethylarsonous acid (MMAIII), and dimethylarsinic acid (DMAV) [28]. Animal studies suggested that dimethylarsinic acid (DMAV) could induce bladder carcinogenesis in rats with the generation of a reactive metabolite (DMA (II I)) [29]. Monomethylarsonous acid (MMAIII)] also induced bladder cell transformation into immortal cells [30]. The relationship between arsenate metabolites and UTUC carcinogenesis in humans remains to be assessed.

There are some limitations to the present study. This retrospective observational study analyzed the clinicopathological features of UTUC patients in BFD non-endemic and endemic areas. Another limitation is that some possible risk factors, such as smoking, were not completely assessed. Nevertheless, it is currently the largest UTUC collaborative group in Taiwan. Additionally, the number of patients in this study was higher than in any previous study.

## Conclusion

In conclusion, there is a slight female predominance in UTUC in Taiwan, which differs from other countries. Clinicopathological profiles of BFD endemic UTUC patients include younger age, previous bladder tumor history, higher pathological grade and stage, more ureteral tumors, less lymphovascular invasion, and unfavorable prognosis, compared to BFD non-endemic UTUC patients. Despite the fact that arsenic intoxication is responsible for the poor prognosis of BFD endemic UTUC patients, more factors should be taken into account in the analyses.

## Data Availability

Medical records were not allowed for the public release.

## Abbreviations

UTUC: Urinary tract urothelial carcinoma
BFD: Black-foot disease
GU: Genitourinary
OS: Overall survival
DFS: Disease-free survival
CSS: Cancer-specific survival
MMAV: Monomethylarsonic acid
MMAIII: Monomethylarsonous acid
DMAV: Dimethylarsinic acid
